# Binder-Free CNT-Modified Excellent Electrodes for All-Vanadium Redox Flow Batteries

**DOI:** 10.3390/nano14090767

**Published:** 2024-04-26

**Authors:** Nitika Devi, Prabhakar Singh, Yong-Song Chen

**Affiliations:** 1 Department of Mechanical Engineering and Advanced Institute of Manufacturing with High-Tech Innovations, National Chung Cheng University, 168 University Rd., Minhsiung Township, Chiayi County 621301, Taiwan; imeysc@ccu.edu.tw; 2 Department of Physics, Indian Institute of Technology, Varanasi 221005, India; psingh.app@iitbhu.ac.in

**Keywords:** carbon nanotube, graphite felt, all-vanadium redox flow battery

## Abstract

Electrodes are one of the key components that influence the performance of all-vanadium redox flow batteries (VRFBs). A porous graphite felt with modified fiber surfaces that can provide a high specific activation surface is preferred as the electrode of a VRFB. In this study, a simple binder-free approach is developed for preparing stable carbon nanotube modified graphite felt electrodes (CNT-GFs). Heat-treated graphite felt electrodes (H-GFs) are dip-coated using CNT homogeneous solution. Cyclic voltammetry (CV) and electrochemical impedance spectroscopy (EIS) results conclude that CNT-GFs have less resistance, better reaction currents, and reversibility as compared to H-GF. Cell performances showed that CNT-GFs significantly improve the performance of a VRFB, especially for the CNT-GF served in the positive side of the VRFB. CNT presence increases the electrochemical properties of the graphite electrode; as a result, reaction kinetics for both VO_2_^+^/VO^2+^ and V^3+^/V^2+^ are improved. Positive CNT-GF (P-CNT-GF) configured VRFB exhibits voltage efficiency, coulombic efficiency, and energy efficiency of 85%, 97%, and 82%, respectively, at the operating current density of 100 mA cm^−2^. At high current density of 200 mA cm^−2^, the VRFB with P-CNT-GF shows 73%, 98%, and 72% of the voltage, coulombic, and energy efficiencies, respectively. The energy efficiency of the CNT-GF is 6% higher when compared with that of B-H-GF. The VRFB with CNT-GF can provide stable performance for 300 cycles at 200 mA cm^−2^.

## 1. Introduction

Redox flow batteries (RFBs) are a type of energy storage device that can store a significant amount of energy from renewable sources. Among various energy storage options, all-vanadium redox flow batteries (VRFBs) stand out as potential candidates due to their safety, high capacity, limited self-discharge, rapid response, long cycle life, and design flexibility. In VRFBs, energy is stored by redox couples V^2+^/V^3+^ and VO^2+^/VO_2_^+^ in the negative and positive electrolyte tanks of the battery system, respectively [1]. An ion exchange membrane is used to separate the positive and negative electrodes of the battery, employing the same element in different oxidation states to minimize cross-contamination through the membrane [2]. Although VRFBs demonstrate more advantages than different types of RFBs, there are still some limitations that need to be addressed, such as low round-trip efficiency compared to Li-ion batteries, and the high cost of vanadium electrolytes. While the electrolyte serves as the storage source for RFBs, electrodes also play a crucial role in defining the performance of the RFBs [3,4].

An electrode plays a crucial role in VRFBs, as its efficiency significantly influences the reaction kinetics. Commonly used electrodes include carbon-based materials such as graphite felts [5,6], carbon fibers [7], and carbon papers [8] due to their high specific surface area and excellent tolerance to extreme chemical conditions. In recent years, polyacrylonitrile (PAN)-based or rayon graphite felts have gained widespread use in VRFB electrodes due to their affordability and high specific porosity. However, pristine graphite felts exhibit low electrochemical performance, prompting the implementation of various strategies such as heat treatment [9,10], chemical treatment [11], and composite formation [12,13] to enhance their functionality.

Heat and chemical treatments primarily involve the introduction of oxygen functional groups, thereby improving the electrocatalytic properties of graphite felts. Additionally, heteroatom doping, as demonstrated by Li et al. [11], who analyzed nitrogen and sulfur co-doped graphene composite graphite felt electrodes for VRFBs, can effectively increase the surface area and functionality of graphite felts. The resulting energy efficiency was reported as 85.37% at a current density of 80 mA cm^−2^ [11]. Phosphorous and oxygen co-doped electrodes, as investigated by another study [12], exhibited a long cycle life with a high discharge capacity of 10.1 Ah L^−1^ at a current density of 350 mA cm^−2^.

Composite formation with metal oxides, such as MnO_2_ [14], ZrO_2_ [15], SnO_2_ [16], and TiO_2_ [17] has also shown promise in improving the reaction kinetics of graphite felt toward vanadium redox couples V^2+^/V^3+^ and VO^2+^/VO_2_^+^. Wu et al. [18] developed PbO_2_-modified graphite felts for the positive electrode through electrodeposition. This economical PbO_2_-modified electrode demonstrated a remarkable 82% energy efficiency at 70 mA cm^−2^. Tang et al. [19] reported a Ti_x_O_y_ modified carbon negative electrode for VRFBs, achieving considerable energy efficiency. At 200 mA cm^−2^, the battery provided an energy efficiency of 82%, and a slight decrease to 77.9% occurred at a higher current density of 260 mA cm^−2^.

Low-dimensional carbon materials, such as graphene (2D), carbon nanotubes (CNT, 1D), and carbon dots (0D), have been recognized for their effectiveness in enhancing electrode performance for VRFBs. Carbon-based materials, known for their high porosity and specific surface area, offer numerous reaction sites. The abundance of carbon further positions it as an economical choice for commercialization, and its high surface reactivity allows for additional modifications to improve performance [20]. Xing et al. [13] employed a template method to prepare a composite electrode consisting of hollow porous carbon spheres decorated on a graphite felt. The unique structure of the carbon sphere, providing active sites both inside and outside the sphere surface, resulted in reduced polarization losses. The VRFB operated at a high current density of 200 mA cm^−2^, delivering an energy efficiency of 82.7% with a peak power density of 958 mW cm^−2^. Similarly, a graphite felt modified with reduced graphene oxide (rGO) was found to be an effective electrode for VRFBs [21]. Lv et al. [22] reported the in situ growth of a carbon network-wrapped graphite felt using electrodeposition. The VRFB achieved an energy efficiency of 79.2% at 100 mA cm^−2^, and even at a high current density of 250 mA cm^−2^, it delivered an energy efficiency of 59.8%.

CNTs have been extensively utilized to enhance the electrochemical properties of electrodes, with reports indicating that both types—single-walled carbon nanotubes (SWCNTs) and multi-walled carbon nanotubes (MWCNTs)—can serve as electrocatalysts for the VO_2_^+^/VO^2+^ and V^3+^/V^2+^ redox couples [23,24]. While CNT-modified carbon electrodes (CNT-GF) have demonstrated excellent electrochemical properties [25], the deposition of CNTs on graphite felt is a challenging task, often requiring the introduction of stabilizing binders like Nafion. Additionally, more complex and sometimes costly techniques, such as chemical vapor deposition, have been developed for implanting CNTs on carbon felts. It is worth noting that many studies have primarily focused on low current densities.

To reduce the electrode cost of VRFBs, a simple, binder-free approach for synthesizing CNT-GFs is proposed in this study. A comprehensive analysis of surface morphology and chemistry using techniques such as field emission scanning electron microscopy (FESEM), contact angle measurements, and Raman spectroscopy was studied and discussed. Electrochemical and impedance spectroscopy analyses were employed to assess the impact of CNT presence on the conductivity of heat-treated graphite felts (H-GFs). The performance of the VRFB, with the CNT-GF utilized in both the anode and cathode, was evaluated at selected current density levels ranging between 100 and 200 mA cm^−2^.

## 2. Materials and Methods

### 2.1. Preparation of CNT-GFs

MWCNTs (Tokyo Chemical Industry Co., Ltd., Tokyo, Japan), with a length of 5–15 μm, diameter of 10–20 nm, and a purity of 97%, were functionalized with acid treatment prior to electrode modification. The CNTs were immersed in a 3:1 solution of HNO_3_ (ACS reagent, 70%, Alfa Aesar, Ward Hill, MA, USA) and H_2_SO_4_ (ACS reagent, 95.0–98.0%, Alfa Aesar) at 90 °C for 24 h. Following this, the CNTs were thoroughly washed with distilled water multiple times to eliminate impurities and achieve a pH-neutral state. The treated CNTs were then dried overnight at 100 °C in a hot air oven.

**Figure 1:**
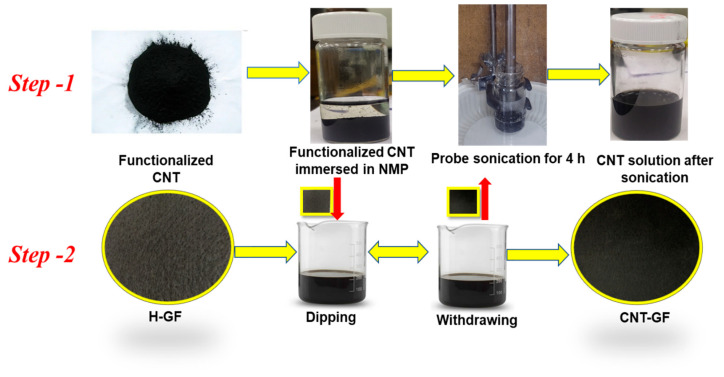
Schematic of CNT-GF preparation.

Subsequently, CNT-graphite felts (CNT-GFs) were prepared using a two-step binder-free process. In the first step, a CNT solution was prepared by dissolving functionalized CNTs in N-Methyl-2-pyrrolidone (NMP) (ACS reagent, 99+%, Alfa Aesar) solvent. Due to the poor solubility of carbon materials in solvents, a 4 h probe sonication was performed to obtain a homogeneous CNT solution. Figure 1 illustrates the described process, highlighting the noticeable difference in the CNT solution before and after probe sonication.

Heat-treated graphite felts (H-GFs) were derived from the heat treatment of pristine graphite felts (GF650, CeTech Co., Ltd., Taichung City, Taiwan) at 500 °C for 6 h in an air atmosphere using a muffle furnace. CNT-GFs were then prepared by dip-coating CNTs onto H-GFs, with a CNT loading of 1 mg cm^−2^. The dip-coated CNT-GFs were left to dry overnight at 80 °C in a vacuum oven.

### 2.2. Characterization of Electrodes

XRD analysis of functionalized CNTs was conducted using a (Rigaku Miniflex, Tokyo, Japan) Cu Kα radiation, covering an angular range from 10° to 85°. Contact angle measurements were performed using a Cam 100 apparatus and analyzed with Creating Nano Technologies Inc. (contact angle meter series, Tainan City, Taiwan). Nitrogen adsorption–desorption isotherms at 77 K were obtained using a Micrometitics ASAP 2020 volumetric adsorption analyzer (Micrometitics ASAP 2020, Norcross, GA, USA). The Barrett–Joyner–Halenda (BJH) approach was applied to obtain the pore-size distribution from the adsorption branch of the relevant isotherm, while the Brunauer–Emmett–Teller (BET) method determined the specific surface area of each sample. The total pore volume was estimated using the amount adsorbed at a relative pressure of P/P0 = 0.99. Raman spectra of the samples were obtained using a LabRAM HR Evol Raman spectrometer (Lab RAM HR, Villeneuve d’ascq, France). The morphology and microstructure of both H-GFs and CNT-GFs were investigated using an FESEM (Hitachi FE-SEM, S-4800, Montreal, QC, Canada).

### 2.3. Evaluation of Electrochemical Performance

Electrochemical measurements of both types of electrodes were conducted using a potentiostat (CHI700E, Austin, TX, USA) in an electrolyte containing 1.684 M vanadium and 4.397 M H_2_SO_4_. Cyclic voltammetry was performed at a scan rate of 5 mV s⁻^1^ within two distinct voltage windows, from −1 to 0 V and from 0 to 2 V. The working electrode area for both H-GF and CNT-GF was maintained at 1 × 1 cm^2^. The electrochemical cell was assembled with Pt serving as the counter electrode and Ag/AgCl filled with 3 M KCl as the reference electrode. Electrochemical impedance spectroscopy (EIS) was carried out using an electrochemical impedance meter (CHI7081D, Austin, TX, USA) with a frequency between 1 kHz and 2 MHz at an applied potential of 0.8 V.

### 2.4. Measurement of VRFB Performance

A VRFB, consisting of a separator, graphite electrodes, graphite plates, gold-coated copper current collectors, and end plates, with an active area of 5 × 5 cm^2^, was used for performance measurement. The separator employed was a commercial membrane (Nafion 212, Dupont, Wilmington, DE, USA). Three different VRFB configurations were studied, each with distinct electrode compositions: B-H-GF (H-GF for both electrodes), P-CNT-GF (CNT-GF for the positive electrode and H-GF for the negative electrode), and N-CNT-GF (CNT-GF for the negative electrode and H-GF for the positive electrode).

Initially, 50 mL of electrolytes composed of 1.684 M VOSO_4_ and 4.397 M H_2_SO_4_ were used for both positive and negative half-cells. The flow rates of both electrodes were maintained at 1.5 L h⁻^1^ for all experiments. The cut-off voltages for the charging and discharging processes were set at 1.69 V and 0.71 V, respectively, with selected current density levels of 100, 150, and 200 cm⁻^2^. Cell retention was also evaluated by applying an initial current density of 100 cm⁻^2^ after operating at a high current density.

## 3. Results and Discussions

### 3.1. Characteristics of CNT-GFs

**Figure 2:**
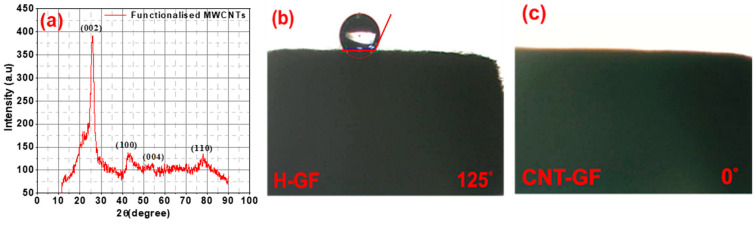
(**a**) XRD of functionalized CNTs, contact angle measurements using 1.684 M vanadium and 4.397 M H_2_SO_4_ electrolyte; (**b**) H-GF; (**c**) CNT-GF.

Figure 2a presents the XRD spectrum of functionalized CNTs, revealing well-defined peaks corresponding to the phases (002), (100), (004), and (110) [26]. Notably, the XRD spectrum of functionalized CNTs closely resembles that of untreated CNTs [26], suggesting that the acid treatment did not significantly alter the structure of the CNTs. In VRFBs, where redox reactions occur on the electrode surfaces, the wettability of the electrode’s surface plays a crucial role in its interaction with the electrolyte.

The surface of a pristine graphite felt is highly hydrophobic, resulting in low wettability and reduced surface energy for redox reactions [27]. Figure 2b,c depicts contact angles for H-GFs at around 125° and CNT-GFs at around 0°. These results indicate that the introduction of CNTs results in a super hydrophilic electrode surface, signifying excellent wettability compared to the surface of an H-GF. This enhanced wettability reduces the impedance of electrode–electrolyte interactions, leading to improved battery reaction kinetics.

**Figure 3:**
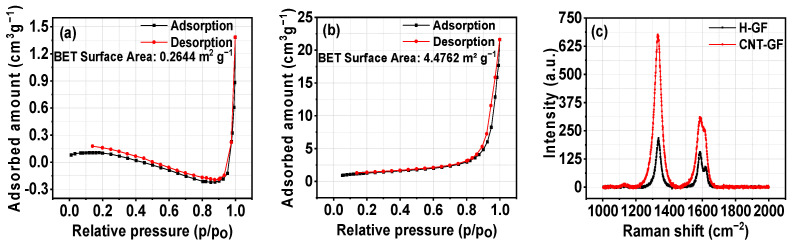
Surface area curves for (**a**) H-GFs; (**b**) CNT-GFs; and (**c**) Raman spectroscopy of H-GFs and CNT-GFs.

Figure 3a,b displays the BET surface area results for H-GFs and CNT-GFs. The BET surface area for CNT-GFs (4.4762 m^2^ g⁻^1^) was significantly higher compared to that of H-GFs (0.2644 m^2^ g⁻^1^), indicating a larger number of reactive sites in CNT-GFs. Aside from surface area, both pore volume and pore diameter exhibited considerable variations in both electrodes. For H-GFs, the pore volume and adsorption average pore diameter were 0.001367 cm³ g⁻^1^ and 20.6726 nm, respectively. In contrast, CNT-GFs showed values of 0.033439 cm³ g⁻^1^ and 29.8811 nm, respectively.

Figure 3c presents the Raman spectrum for H-GFs and CNT-GFs. The spectrum reveals an increased intensity of both G and D bands in the presence of CNTs. The G band corresponds to the stretching vibration of sp2-bonded carbon around 1600 cm⁻^1^, while the D band is related to sp3-bonded vibrations at 1340 cm⁻^1^ [28]. The D band signifies the presence of defect sites, and the I_D_/I_G_ ratio, which was 1.40 for H-GF and 2.20 for CNT-GF, indicates a higher degree of disorder in carbon materials in CNT-GFs. This higher degree of disorder is favourable for vanadium ion reactions, as it provides more defect sites.

**Figure 4:**
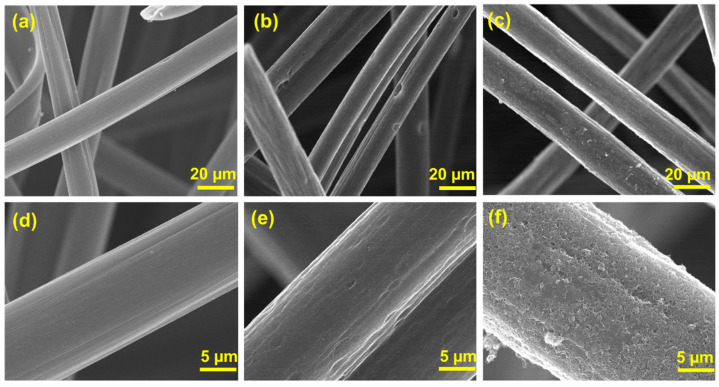
FESEM surface morphology images of (**a**,**d**) pristine graphite felt; (**b**,**e**) H-GF; (**c**,**f**) CNT-GF.

The FESEM images in Figure 4 illustrate the morphological differences between the surfaces of H-GF and CNT-GF. Figure 4a,b presents the FESEM image of the pristine graphite felt, while Figure 4c,d shows the FESEM image of H-GF after heat treatment. It can be observed that there is no significant difference in morphology between the pristine graphite felt and H-GF after heat treatment. The heat treatment was specifically performed to enhance the reactive sites of the graphite felt without causing damage to its surface morphology.

Figure 4e,f depicts the surface of CNT-GF, which appears less smooth compared to the pristine graphite felt and H-GF due to the presence of CNTs. Notably, Figure 4f illustrates the successful and uniform distribution of CNTs on the graphite felt surface.

### 3.2. Electrochemical Characteristics of CNT-GFs

**Figure 5:**
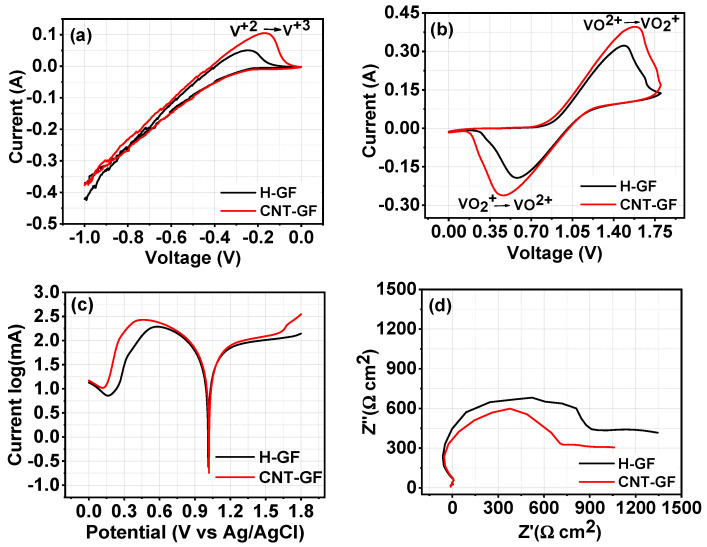
CV of H-GF, CNT-GF (**a**) for voltage window −1–0; (**b**) 0–2; (**c**) EIS measurement of H-GF and CNT-GF in 1.684 M VOSO_4_ + 4.397 M H_2_SO_4_ with reference to Ag/AgCl; (**d**) electrochemical impedance spectroscopy (EIS) results.

This study focuses on evaluating the performance of CNT-GF as the electrode for VRFB, with electrochemical characteristics analyzed by cyclic voltammetry (CV) in two different voltage windows. The voltage windows used were between −1 and 0 V (vs. SCE) for analyzing V^2+^/V^3+^ redox reactions, and between 0 and 2 V (vs. SCE) for VO^2+^/VO_2_^+^ redox reactions. Figure 5 illustrates the CV curves for both types of redox pairs, Tafel plots, and provides a comparison of the electrode–electrolyte interface resistance for both types of electrodes. Table 1 lists the Ipc and Ipa currents along with their reduction and oxidation potentials for VO^2+^/VO_2_^+^.

In the case of V^2+^/V^3+^, the currents were not determined, as the reaction did not show a clear reversible nature, as shown in Figure 5a. This irreversibility may be because of VO^2+^ and V^3+^ reaction on the electrode surface which decreased the concentration of V^3+^. VO^2+^/V^3+^ couple is thermodynamically favourable with a theoretical cell voltage of 0.66 V, which is usually considered as self-discharge reaction in VRFB [29]. On the contrary, VO^2+^/VO_2_^+^ showed obvious reversible CV curves for both H-GFs and CNT-GFs, as shown in Figure 5b. Table 1 summarizes that both the cathodic (−*I*_pc_) and anodic (*I*_pa_) currents of H-GFs (−*I*_pc_: 0.193 A, *I*_pa_: 0.322 A) improved to −*I*_pc_: 0.263 A and *I*_pa_: 0.396 A in CNT-GF, where the H-GF was modified by CNTs. These results are consistent with the literature, which showed that the conductivity of GF electrodes increased with the surface modification of the electrodes with conducting and high surface area materials [30]. Here, CNT presence enhances the electrochemical properties of GF electrodes due to CNT conductivity and surface area contributions. These results are evident from the increased current response of CNT-GF electrodes in both oxidation and reduction reactions. Further, −*I*_pc_/*I*_pa_ and Δ*E*(V) were studied to obtain a deep insight into chemical reactions for analyzing the reaction reversibility and feasibility, respectively [31]. The ratio of −*I*_pc_/*I*_pa_ indicates that redox reactions were more reversible in the case of CNT-GFs, with its value closer to 1. Surprisingly, Δ*E*(V), the separation of oxidation and reduction reactions, is slightly greater in CNT-GF (1.11 V) than in H-GF (0.98 V), which may be due to the high surface area of CNT-GF electrodes, which is responsible for increased CV area under the curve. In V^2+^/V^3+^, a clear increase in the oxidation current can be seen in CNT-GF. Additionally, in both types of reactions, the area under the CV curve increases in CNT-GF, indicating better electrochemical behavior. This behavior is typical in carbon materials, as the high surface area and porosity is the main contributor to good electrochemical performance [32]. In surface area results, CNT-GF has more surface area and porosity, which contribute to easy adsorption–desorption of vanadium ions, thus improving the storage energy. Thus, CNT improves the electrochemical behavior of H-GFs.

To obtain a deeper understanding of reaction kinetics, Tafel plot analysis was carried out to calculate the exchange current densities and electron transfer coefficients from CV data of VO^2+^/VO_2_^+^. Tafel plots of H-GF and CNT-GF electrodes are shown in Figure 5c, and analysis was performed using the Butler–Volmer equation supporting the Tafel behavior. The following equations were used to compute the intercept and slope parameters

nact=a+b ln⁡Io 

$$a=-\frac{RT}{\alpha F}\ln\left(I_{o}\right)\;,\;b=\frac{RT}{\alpha F}$$

Here, *a*, and *b* are Tafel intercept and Tafel slope, respectively. *n_act_* are the activation losses, *R* is universal gas constant, *T* is temperature, *F* is Faraday constant, $I_{o}$ is exchange current density, and *α* is electron transfer coefficient [33]. Cathodic and anodic exchange current densities (Ioc, Ioa (mA cm^−2^)), and cathodic and anodic electron transfer coefficient (*α*_c_, *α*_a_) for both H-GF and CNT-GF electrodes are reported in Table 2. Tafel slopes and intercept were used for calculating cathodic and anodic exchange current densities and electron transfer coefficients. The behavior of these parameters was consistent with the published reports, which showed that increased surface-active sites result in higher increased exchange current density and increased electron transfer coefficient [34]. Electron transfer coefficient signifies the electrochemical reaction kinetics by catalytic activity, and exchange current density represents the electrochemical current rate produced by reactions [33]. These two parameters were increased in the case of CNT-GF electrode, which indicates improved reaction kinetics. Cathodic and anodic exchange current densities for CNT-GF were 1.27 and 3.12 mA cm^−2^, respectively, which were 1.22 and 3.03 mA cm^−2^ for H-GF electrode. These findings suggest that CNT modification enhanced electrode–electrolyte reaction kinetics.

Furthermore, electrochemical impedance spectroscopy (EIS) results in Figure 5d show that CNT-GFs have a smaller impedance compared to H-GF due to an increase in the conductivity of H-GF resulting from the presence of conductive CNTs. These results are consistent with the enhanced electrochemical CV results. Additionally, impedance of CNT-GF electrode was less as compared to H-GF, which means that electrode–electrolyte interactions were improved with CNT presence, which was also evident from contact angle measurements. Surface kinetics of CNT-GF electrode improved due to CNT high surface area and conductivity, which decreased the electrode–electrolyte resistance.

### 3.3. VRFB Performance

**Figure 6:**
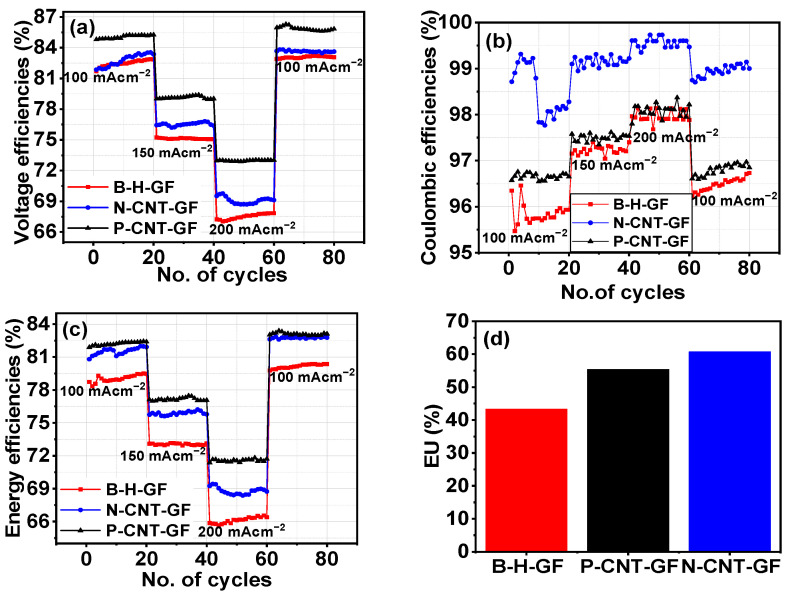
(**a**) Voltage efficiencies; (**b**) coulombic efficiencies; (**c**) energy efficiencies for B-H-GF and CNT-GF as negative (N-CNT-GF) and positive electrode (P-CNT-GF); (**d**) a comparison of electrolyte utilization (EU) for B-H-GF, P-CNT-GF, and N-CNT-GF VRFBs.

The performance of VRFBs with different GF electrodes is presented in Figure 6. The VRFB with CNT-GFs exhibited higher voltage, coulombic, and energy efficiencies compared to the VRFB with B-H-GFs at three different current densities. Moreover, the performance of VRFBs with either P-CNT-GF or N-CNT-GF surpassed that of H-GF. Specifically, the VRFB with P-CNT-GF demonstrated higher voltage efficiencies without compromising coulombic efficiencies, attributed to the increased conductivity provided by CNTs, reducing the cell’s polarization.

However, N-CNT-GF showed superior coulombic efficiencies compared to the other two VRFBs and higher voltage efficiencies than B-H-GF. This improvement in coulombic efficiencies can be attributed to the higher electrode reversibility of the VRFB. Voltage efficiency improvement in both P-CNT-GF and N-CNT-GF was due to increased conductivity of the CNT-GF electrode and decreased electrode–electrolyte resistance as obtained in the electrochemical study. As energy efficiencies are the product of voltage efficiencies and coulombic efficiencies, the energy efficiencies were higher in both VRFBs consisting of CNT-GFs [35]. At an operating current density of 100 mA cm⁻^2^, the energy efficiency was 79%, 81%, and 82% for B-H-GF, N-CNT-GF, and P-CNT-GF, respectively. The impact of CNTs was more pronounced at high current densities of 200 mA cm⁻^2^, where energy efficiencies were 66%, 69%, and 72% for B-H-GF, N-CNT-GF, and P-CNT-GF, respectively. This indicated that the energy efficiency of P-CNT-GF VRFB increased by 6% compared to the VRFB with B-H-GFs at high current density. This means that even at higher current densities, cell polarization did not become prominent in the case of N-CNT-GF, and P-CNT-GF, which was responsible for decreasing the performance of B-H-GFs at high current densities.

Interestingly, electrolyte utilization (EU) showed a significant improvement in both N-CNT-GF and P-CNT-GF compared to B-H-GF. The average EU for B-H-GF was around 43%, while it increased to around 58% and 61% for P-CNT-GF and N-CNT-GF, respectively, representing an improvement of more than 15%. This enhanced EU is consistent with the literature [36], which demonstrates that improved electrode properties also enhance EU. Table 3 provides a detailed summary of values of voltage efficiencies, coulombic efficiencies, and energy efficiencies at current densities of 100, 150, and 200 mA cm⁻^2^. The trend indicates that efficiencies decrease with increasing current densities, attributed to larger charge–discharge overpotential at high currents [37]. VRFB retention was evaluated by applying a low current density of 100 mA cm⁻^2^ after operating at higher current densities, with all VRFBs showing 100% retention.

**Figure 7:**
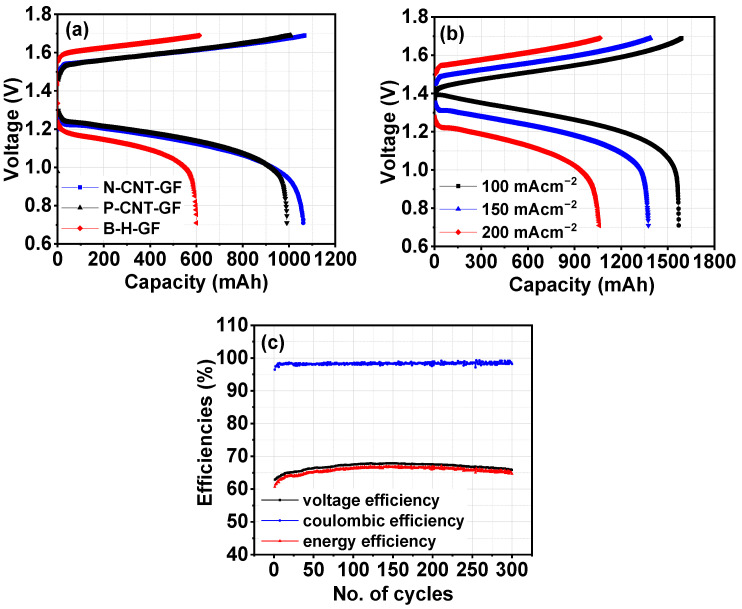
(**a**) Comparison of first cycle charge–discharge capacity of all three types of VRFB configurations using B-H-GF and CNT-GF at 200 mA cm^−2^ current density; (**b**) comparison of first cycle charge–discharge capacity of N-CNT-GF VRFB configuration at three different current densities of 100, 150, 200 mA cm^−2^; (**c**) P-CNT-GF VRFB performance at 200 mA cm^−2^ for 300 charge–discharge cycles.

In Figure 7a, a comparison of charge–discharge capacity at 200 mA cm⁻^2^ in the first cycle for all VRFBs is presented. Both charge and discharge capacity significantly improved for P-CNT-GF and N-CNT-GF. This capacity improvement is attributed to reduced polarization losses in CNT-GF compared to H-GF. Figure 7b demonstrates the effect of charge–discharge capacity with varying current densities for P-CNT-GF, showing a decrease in capacity with an increase in current density. This observation aligns with published reports, where an increase in current density results in larger charge–discharge polarization losses [37]. Thus, increased current density results in decreased VRFB performance. Table 3 lists the VRFB’s performance at various current densities. It can be reflected that increased current density results in decreased energy efficiencies. However, the effect was less prominent in the case of P-CNT-GF and N-CNT-GF due to improved electrochemical behavior of CNT-GF. P-CNT-GF showed the best performance among other VRFBs, which was probably due to the increased influence of CNT-GF kinetics toward the VO^2+^/VO_2_^+^ redox pair, as evident from electrochemical measurements.

Since P-CNT-GF showed the best energy efficiencies, this VRFB was further evaluated for 300 continuous charge–discharge cycles at a higher current density of 200 mA cm⁻^2^. All efficiencies are displayed in Figure 7c, revealing stable performance over 300 cycles. Stable efficiencies indicates that CNT presence was stable throughout the 300 charge–discharge cycles. Table 4 offers a comprehensive comparison of the outcomes obtained in this study with those reported in similar studies involving CNT modification of carbon felt for VRFB applications. Performance achieved in this work is considerably higher in comparison to other reported works, which can be attributed to binder-free and uniform deposition of CNT. This comparative analysis underscores the success of the presented simple binder-free approach in preparing a CNT-GF electrode, which proves to be capable of delivering significant and promising battery performance.

## 4. Conclusions

The redox reaction of a VRFB occurs on the electrode/electrolyte interface; thus, reducing interface impedance is an important factor in improving VRFB performance. In this study, a CNT-GF is developed to enhance the performance of a VRFB. The characteristics and electrochemical properties of the CNT-GFs are studied, and the performance of VRFBs with CNT-GFs is investigated. The following conclusions can be drawn from the study.

1. A CNT homogeneous solution was successfully prepared using NMP solvent with the aid of probe sonication, followed by the dip-coating method to coat CNTs on H-GF. FESEM and Raman analysis confirmed the successful deposition of CNTs on H-GF, resulting in improved wettability, as demonstrated by super hydrophilic behavior.
2. The CNT-GF exhibited lower resistance and higher cathodic and anodic peak currents for both vanadium redox pairs. These results indicate enhanced reaction kinetics of VO^2+^/VO_2_^+^ and V^3+^/V^2+^ whether the CNT-GF serves in the positive or negative side of a VRFB.
3. At a current density of 100 mA cm^−2^, VRFBs with N-CNT-GF or P-CNT-GF demonstrated energy efficiencies of 81% or 82%, respectively, whereas the VRFB with B-H-GFs showed an energy efficiency of 79%. At a high current density of 200 mA cm^−2^, P-CNT-GF delivered an energy efficiency of 72%, a 6% improvement over B-H-GF. This improvement is attributed to lower polarization losses and higher electrode reversibility.
4. Coating CNTs on H-GFs improved the electrolyte utilization of the VRFB, increasing it from 43% to around 60%, a significant improvement of 17%.
5. The VRFB exhibited stable operation for 300 cycles at 200 mA cm^−2^, demonstrating the durability of the CNT-GF electrode.

## Figures and Tables

**Table 1:** Summary of *I*a*, I*c*, E*a*, E*c*,* and Δ*E* calculated from VO^2+^/VO_2_^+^ CV for H-GF and CNT-GF.

Electrode Type	−*I*_pc_ (A)	*I*_pa_ (A)	−*I*_pc_/*I*_pa_	*E*_r_ (V)	*E*_o_ (V)	*E* (ΔV)
H-GF	0.193	0.322	0.59	0.54	1.52	0.98
CNT-GF	0.263	0.396	0.66	0.46	1.57	1.11

**Table 2:** Reaction kinetics parameters obtained from Tafel plot.

Electrode Type	Ioc (mA cm^−2^)	Ioa (mA cm^−2^)	*α*_c_	*α*_a_
H-GF	1.22	3.03	0.07	0.052
CNT-GF	1.27	3.12	0.11	0.056

**Table 3:** Detail of voltage efficiencies, coulombic efficiencies, and energy efficiencies for all three kind of cell configurations at current densities 100, 150, 200 mA cm^−2^ and reverse current density 100 mA cm^−2^.

Current Density	Voltage Efficiency	Coulombic Efficiency	Energy Efficiency
(mA cm^−2^)	(%)	(%)	(%)
B-H-GF			
100	82.48	95.85	79.05
150	75.13	97.23	73.05
200	67.5	97.97	66.13
100	83.10	96.48	80.16
N-CNT-GF			
100	82.78	98.49	81.53
150	76.52	99.15	75.87
200	69.08	99.57	68.78
100	83.65	98.95	82.76
P-CNT-GF			
100	85.06	96.65	82.21
150	79.13	97.49	77.16
200	72.97	98.09	71.57
100	85.85	96.80	83.10

**Table 4:** VRFB performance comparison with previously reported CNT-modified electrodes.

No.	Electrode	Cell Size (cm^2^)	Electrolyte	Current Density (mA cm^−2^)	Energy Efficiency (%)	Ref.
1.	HAA-CNT ^1^	4 × 4	1.5 M VOSO_4_ + 2 M H_2_SO_4_	120	77.5	[38]
2.	Nitrogen doped—CNT/CF	6 × 8	0.9 M V(III) + 0.8 M V(IV) + 2 M H_2_SO_4_	40	76.3	[39]
3.	B-CNT/TA-GF ^2^	5 × 5	5 mM V_2_SO_4_ in 2 M	80	76.8	[40]
4.	CNT-GF	5 × 5	2 M VOSO_4_ + 3 M H_2_SO_4_	50	86.9	[41]
5.	Nitrogen doped CNT/GF(Fe)	3 × 3	0.1 M VOSO_4_ + 3.0 M H_2_SO_4_	150	69	[24]
6.	MWCNTs	6 × 6	1.5 M VOSO_4_ + 2 M H_2_SO_4_	50	82	[35]
7.	P-CNT-GF	5 × 5	1.684 M VOSO_4_ + 4.397 M H_2_SO_4_	100	82	This work
8.	P-CNT-GF	5 × 5	1.684 M VOSO_4_ + 4.397 M H_2_SO_4_	150	77	This work
9.	P-CNT-GF	5 × 5	1.684 M VOSO_4_ + 4.397 M H_2_SO_4_	200	72	This work

^1^ HHA-CNT: hydroxamic acid functionalized carbon nanotube; ^2^ B-CNT/TA-GF: bamboo-CNT/Thermal acid-GF.

## Data Availability

The data presented in this study are available on request from the corresponding author.
